# Unmasking Visceral Leishmaniasis: Nephrotic Syndrome as a Rare Pediatric Complication in Morocco

**DOI:** 10.7759/cureus.82540

**Published:** 2025-04-18

**Authors:** Ghizlane Kassal, Houda Nassih, Rabiy Elqadiry, Aicha Bourrahouat, Imane Aitsab

**Affiliations:** 1 Department of Pediatrics, University Hospital Center Mohammed VI, Marrakech, MAR

**Keywords:** children, hepatomegaly, morocco, nephrotic syndrome, splenomegaly, visceral leishmaniasis

## Abstract

Nephrotic syndrome in children with visceral leishmaniasis (VL) is an uncommon presentation, often accompanied by other clinical signs such as febrile splenomegaly. This report highlights two cases of VL in immunocompetent children admitted to Mohamed VI University Hospital in Marrakech, who developed nephrotic syndrome as a complication. One child unfortunately passed away, while the other responded well to treatment with corticosteroids and pentavalent antimonial drugs, showing favorable clinical progress. This rare condition underscores the need to raise awareness among clinicians to facilitate early diagnosis and prompt treatment.

## Introduction

Leishmaniasis is a vector-borne tropical disease caused by intracellular protozoa of the genus *Leishmania*. Its clinical spectrum varies from self-limiting cutaneous ulcers to severe systemic, multi-organ involvement. The disease manifests primarily as visceral leishmaniasis (VL, or kala-azar), cutaneous leishmaniasis (CL), and mucocutaneous leishmaniasis (MCL). VL affects 0.2-0.4 million people annually [1,2]. The definitive diagnosis of VL relies on the identification of the parasite in samples obtained from bone marrow, spleen, or lymph nodes, which remains the diagnostic gold standard.

Renal impairment may result from various factors, including anemia, hypoalbuminemia, hypovolemia, and direct renal toxicity. Histological analysis often reveals changes such as tubulointerstitial nephritis, proliferative glomerulonephritis, and proximal tubulopathy [3,4]. Severe nephrotic-range proteinuria in VL has been reported exclusively in cases associated with either HIV or amyloidosis [4].

We report two cases of nephrotic syndrome as a complication of VL in immunocompetent children. These cases were managed in the Pediatric Department at Mohamed VI University Hospital in Marrakech, a public healthcare institution in Morocco. To the best of our knowledge, these represent the first documented cases of their kind in Morocco.

## Case presentation

Case 1

It’s about a 10-year-old child from Ouarzazate, a Moroccan city known for its endemic leishmaniasis. The child was admitted with fever and hemorrhagic syndrome persisting for about a month. There was no history of recurrent infections or chronic diarrhea. Clinical examination revealed hepato-splenomegaly (HPSM) and a left leg ulcer measuring 5 cm × 4 cm. The child weighed 32 kg and measured 140 cm in height, both within the mean standard deviations. Edema was noted in the lower limbs and facial puffiness.

**Figure 1:**
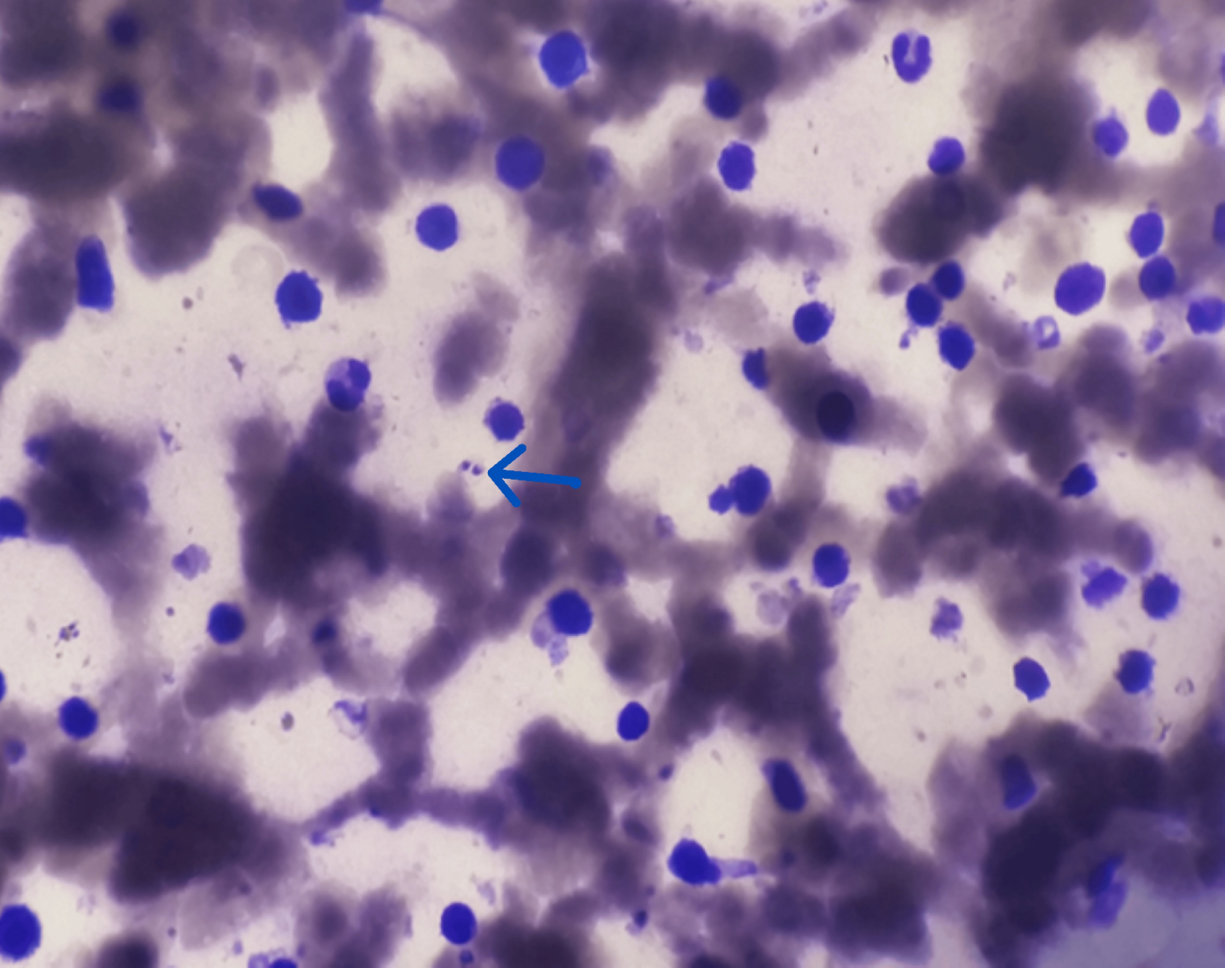
Bone Marrow Aspiration Showing Leishmania Amastigotes (Blue Arrow)

A urine dipstick (UD) test indicated massive proteinuria (+++), without hematuria. Nephrotic syndrome was confirmed with hypoalbuminemia (18.6 g/L) and 24-hour proteinuria (63.75 mg/kg/24 hours). Pancytopenia was observed in the blood count, and bone marrow aspiration confirmed the presence of *Leishmania* amastigotes (Figure 1 and Table 1).

**Table 1:** Summary of Laboratory Findings for Case 1 and Case 2 VL: Visceral leishmaniasis; MAS: Macrophage activation syndrome; NS: Nephrotic syndrome; TP: Total protein; GPT: Serum glutamic pyruvic transaminase; GOT: Serum glutamic oxaloacetic transaminase; LDH: Lactate dehydrogenase; GFR: Glomerular filtration rate; Uprot/Ucr: Urine protein-to-creatinine ratio; ND: Not done

Variables	Case 1-Child: (VL+MAS+NS)		Case 2-Infant: (VL+NS)	
	Initial assessment	Day 5	Initial assessment	Day 7
Leukocytes	1300 (7000-13000)	2100	1500 (3800-10400)	3000
Hemoglobin (Hb, g/dL)	6.3 (10-12)	7.5	5.3 (11-14)	12
Platelets	30000 (185000-399000)	40000	45000 (150000-450000)	139000
Albumin (g/L)	18.6 (25-49)	23	32 (35-48)	13
TP (%)	80 (70-100)	30	90 (70-100)	100
GPT (U/l)	35 (9-25)	850	35 (9-25)	40
GOT (U/l)	46 (21-44)	973	40 (18-36)	25
Ferritin (ng/mL)	100 (5.3-99.9)	833	750 (13.7-78.8)	150
Triglyceride (mg/dL)	6.1 (25-119)	365	200 (22-131)	30
Fibrinogen (g/L)	2.0 (1.57-4.0)	<1.1	2.5 (1.7-4.09)	3.2
LDH (U/L)	3.8 (192-321)	700	411 (170-283)	195
GFR (mL/min)	140 (95-150)	51	140 (95-150)	120
24-hour proteinuria (mg/kg/24h)	63.75 (<5)	-	ND	ND
Uprot/Ucr ratio	ND	ND	ND	3.1 (<0.2)

HIV serology was negative. Treatment with Glucantime at 60 mg/kg/day was initiated, leading to a gradual decrease in the edematous syndrome.

However, after five days, the patient developed respiratory distress, acute renal failure (glomerular filtration rate: GFR 51 mL/min), and hepatocellular failure, prompting discontinuation of Glucantime. Macrophage activation syndrome (MAS) was diagnosed based on fever, splenomegaly, pancytopenia, hypertriglyceridemia (365 mg/dL), hypofibrinogenemia (≤1.1 g/L), and hyperferritinemia (833 u/ml) (Table 1). Methylprednisolone boluses (1 g/1.73 m^2^/day) and oral corticosteroid therapy were administered. Despite adequate management, the patient’s condition worsened, leading to multivisceral failure and death on the 15th day of hospitalization.

Case 2

**Figure 2:**
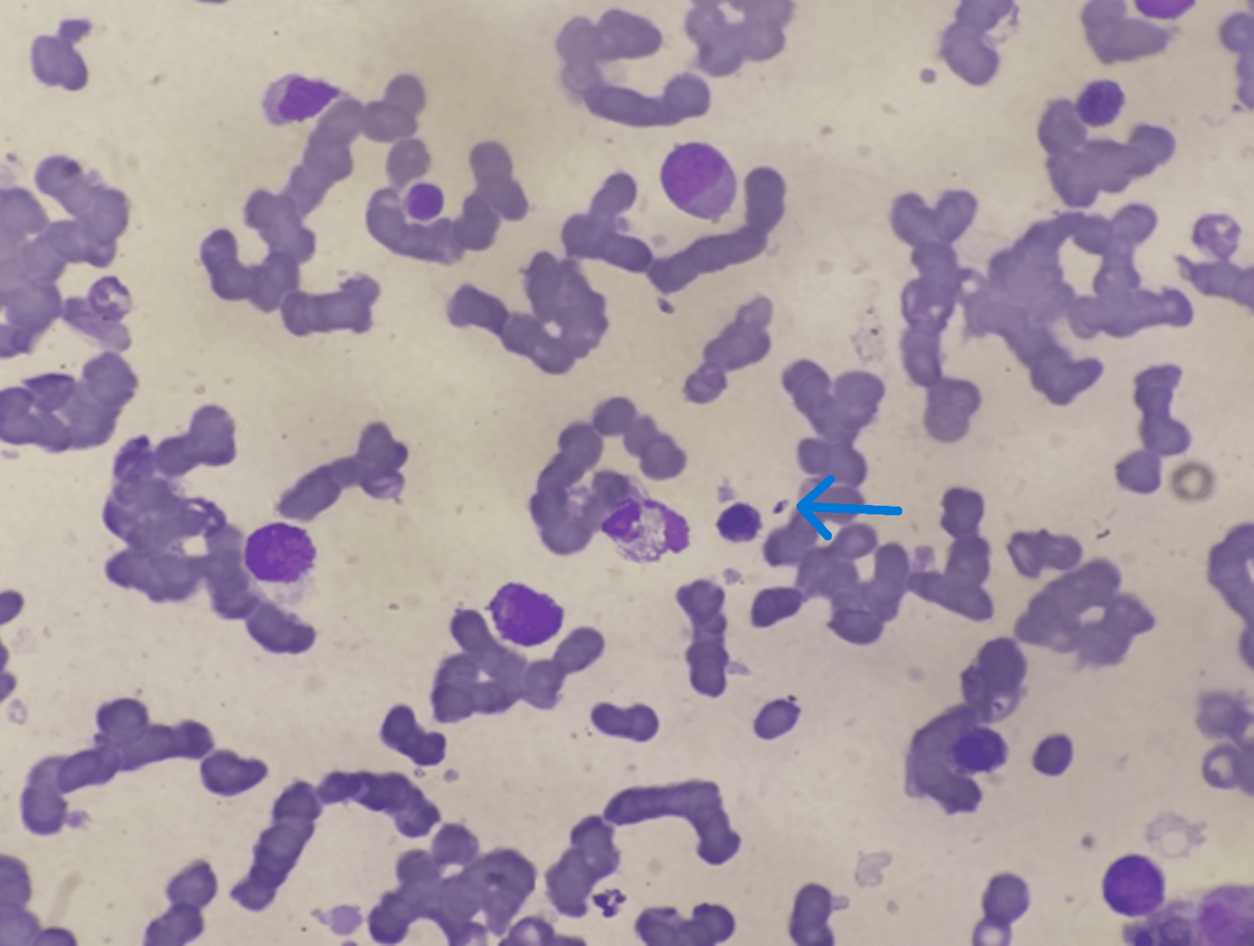
Bone Marrow Aspiration Showing Leishmania Amastigotes (Blue Arrow)

A two year and six months old child, without a pathological history, residing in Ouarzazate, was admitted with a significant decline in general health following a prolonged six-month fever. He weighed 13 kg (below -1 SD) and measured 92 cm in height (mean SD). Clinical and biological assessments revealed HPSM and pancytopenia, with a GFR of 140 mL/min. Serological tests and a myelogram confirmed VL (Figure 2).

**Figure 3:**
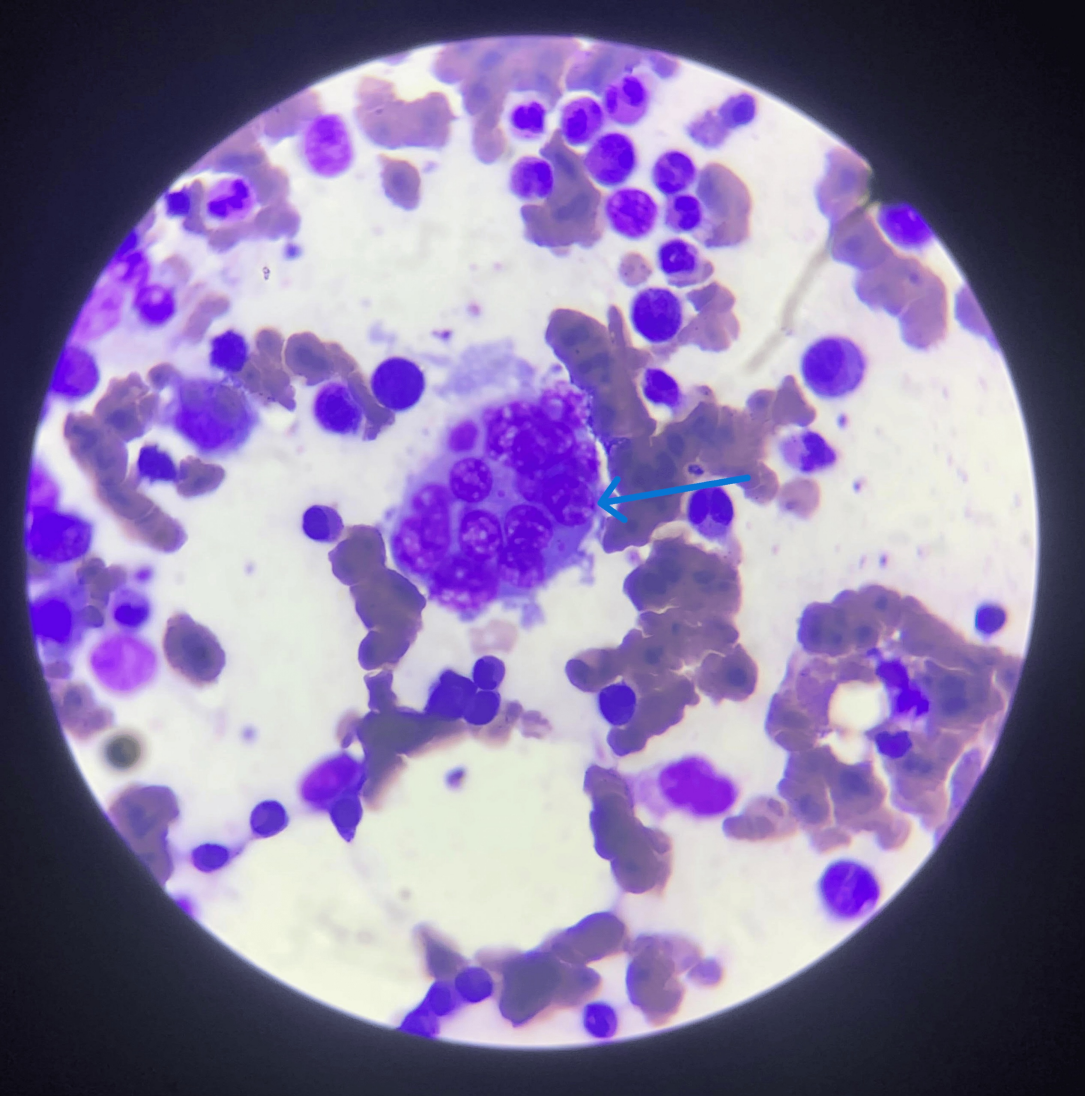
Bone Marrow Aspiration Showing Hemophagocytic Activity (Blue Arrow)

The presence of hemophagocytosis, along with elevated lactate dehydrogenase (LDH) (411 U/L) and ferritin (750 U/L) levels, led to the diagnosis of associated MAS (Table 1, Figure 3, and Appendix A).

Treatment consisted of three intravenous boluses of methylprednisolone (1 g/1.73 m^2^/day), followed by oral corticosteroid therapy, resulting in rapid clinical improvement. Glucantime was started as early as the seventh day after corticosteroid therapy.

In addition, the child developed peripheral edema with ascites and proteinuria confirmed using the UD. Nephrotic syndrome was diagnosed based on hypoalbuminemia (13 g/L) and a urinary protein-to-creatinine (Uprot/Ucr) ratio of 3.1 g/g. HIV serology was negative. The child was treated with an albumin infusion, resulting in a favorable clinical outcome, with proteinuria resolving entirely after 28 days of therapy. No relapse was observed during a one-year follow-up period.

## Discussion

Leishmaniasis occurs not only in tropical regions but also in semi-tropical areas. Over 90% of VL cases are concentrated in six countries. VL primarily affects children and is a life-threatening disease if left untreated [1,2]. In Morocco, as well as in Algeria and Tunisia, VL is predominantly caused by *Leishmania infantum* (90%). Clinically, it manifests as a febrile illness associated with weight loss, pancytopenia, hepatosplenomegaly, and lymphadenopathy, with potential complications such as acute renal damage [5-7]. Renal involvement can encompass both tubular and glomerular dysfunction, resulting in proteinuria and hematuria [8,9]. Massive proteinuria can be a defining feature of nephrotic syndrome, which is clinically characterized by the presence of edema, hypoalbuminemia (serum albumin <25 g/L), and nephrotic-range proteinuria, typically defined as ≥40 mg/m^2^/hour or a Uprot/Ucr ratio exceeding 200 mg/mmol in a spot urine sample [10].

Glomerulopathy in VL is thought to result from immune complex deposition, driven by the polyclonal activation of B lymphocytes induced by the parasite. However, research suggests that other immune system components, such as macrophages, T cells, and cytokines, may play a more significant role than immune complexes in certain cases [11]. Additionally, Leishmania parasites can directly invade kidney tissue, causing localized damage [12].

As noted in the literature, the two cases described in this study illustrate nephrotic syndrome as a rare complication of VL in children. Pentavalent antimony salts remain the most widely used treatment for VL; however, their nephrotoxic potential increases in cases of overdose or impaired renal function. Liposomal amphotericin B (AmBisome) offers a less nephrotoxic alternative and enhances intramacrophagic penetration of antileishmanial therapies.

The second case had severe hepatic impairment, such as hepatocellular insufficiency, alongside renal impairment, which made the treatment available in our context (Glucantime) impossible. In addition, due to the lack of availability of AmBisome, no treatment was established. And severe thrombocytopenia further complicated, making renal puncture biopsy impossible.

High-dose corticosteroid therapy combined with antileishmanial agents appears to be crucial in managing severe renal impairment, especially when its origin is immune-mediated. This therapeutic approach was effectively administered to the patients in this study.

## Conclusions

VL is an uncommon yet notable cause of secondary nephrotic syndrome in children. Its potential should be particularly considered for pediatric patients residing in endemic regions. Raising clinicians’ awareness of this condition and its possible nephrotic complications is crucial, as it enables timely diagnosis and appropriate treatment, ultimately improving patient outcomes.

## Appendices

Appendix A 

**Table 2:** HLH-2004 Criteria [13], 2016 MAS Classification Criteria [14], and HScore [15] ᵃ Fever and splenomegaly are common in patients with sJIA. ᵇ Organ dysfunction is a principal feature of MAS but is not included in the MAS diagnostic tools. MAS: Macrophage activation syndrome; HLH: Hemophagocytic lymphohistiocytosis; HScore: Hemophagocytic syndrome diagnostic score; CBC: Complete blood cell count; AST: Aspartate aminotransferase; TG: Triglycerides; APRs: Acute phase reactants; sIL-2R: Soluble interleukin-2 receptor; NK: Natural killer; BM: Bone marrow; LN: Lymph node; IAHS: Infection-associated hemophagocytic syndrome; sJIA: systemic juvenile idiopathic arthritis

	HLH-2004	2016 MAS Classification	HScore (Points)
Fever	≥38.5 °C	Feverᵃ	38.4-39.4 °C (33); > 39.4 °C (49)
Organomegaly	Splenomegaly	Splenomegalyᵃ	Hepato- or splenomegaly (23); both (38)
Abnormal CBC	Cytopenia ≥ 2 cell lines	Platelet counts ≤ 181,000/µL	2 cell lines (24); 3 cell lines (34)
Elevated liver enzymes	(Supportive evidence)	AST > 48 U/L	AST ≥ 30 U/L (19)
Abnormal TG	TG ≥ 265 mg/dL	TG > 156 mg/dL	133-354 mg/dL (44); >354 mg/dL (64)
Fibrinogen	Fibrinogen ≤ 150 mg/dL	Fibrinogen ≤ 360 mg/dL	≤ 250 mg/dL (30)
Hyperferritinemia	Ferritin ≥ 500 ng/mL	Ferritin > 684 ng/mL	2000-6000 ng/mL (35); > 6000 ng/mL (50)
Elevated cytokines/APRs	sCD25 (sIL-2R) ≥ 2400 U/mL	-	-
Changes in NK cell activity	Low NK cell function	-	-
Hemophagocytosis	BM or LNs	-	-
Infectious triggers	(e.g., IAHS)	(Frequent in many cases)	Known immunosuppression (18)
Organ dysfunctionᵇ	-	-	-
Diagnosis	≥ 5/8 criteria	High ferritin + ≥ 2/4 criteria	Sum of points ≥ 169
